# Supplementary material for: Characterizing the properties of bisulfite sequencing data: maximizing power and sensitivity to identify between-group differences in DNA methylation
Source: BMC Genomics. 2021 Jun 15;22:446. doi: 10.1186/s12864-021-07721-z (PMC8204428; doi:10.1186/s12864-021-07721-z)
Supplement: Supplementary file 1 — Additional file 1: Supplementary Figure 1. The distribution of DNAm levels across the genome profiled using RRBS or a custom array. Supplementary Figure 2. DNAm estimates derived from RRBS are on average lower than those from the array platform. Supplementary Figure 3. A histogram of POWEREDBiSeq calculations showing variability in estimated power. Supplementary Figure 4. r is more accurately estimated when using a larger number of DNAm sites. Supplementary Figure 5. DNAm priors are more accurately estimated when using more DNAm sites. Supplementary Figure 6. The proportion of DNAm sites remaining is more accurately estimated when using more DNAm sites. Supplementary Table 1. A summary of parameters used in simulation analysis. [file 12864_2021_7721_MOESM1_ESM.docx]

**Characterizing the properties of bisulfite sequencing data: maximizing power and sensitivity to identify between-group differences in DNA methylation**

Dorothea Seiler Vellame^1^, Isabel Castanho^1^, Aisha Dahir^1^, Jonathan Mill^1*^, Eilis Hannon^1*^

1 College of Medicine and Health, University of Exeter, Royal Devon and Exeter Hospital, Exeter EX2 5DW, UK.

*These authors contributed equally to this work

**Supplementary Figures**

**Supplementary Figure 1: The distribution of DNAm levels across the genome profiled using RRBS or a custom array.** Density plot of the distribution of DNAm level across all DNAm sites, where each line represents one of the 80 overlapping samples profiled using a custom Illumina array [1] (blue)or RRBS (green). ****

**Supplementary Figure 2: DNAm estimates derived from RRBS are on average lower than those from the array platform.** Shown are boxplots of the difference in DNAm estimated from the Illumina mammalian array and RRBS DNAm data across all overlapping DNAm points, with DNAm points grouped based on their read depths in the RRBS data. ****

**Supplementary Figure 3: A histogram of POWEREDBiSeq calculations showing variability in estimated power.** The estimated power of a study was calculated 400 times, using a mean DNAm difference between groups of 0.06 and minimum sample size of 60. Users can calculate bespoke power estimates using their own study-specific parameters in POWEREDBiSeq.

**Supplementary Figure 4: r is more accurately estimated when using a larger number of DNAm sites.** The negative binomial parameter, r, was calculated from an increasing number of DNAm sites using RRBS data from 125 samples. The red dashed line is the true value of r, calculated from the entire dataset. ****

**Supplementary Figure 5: DNAm priors are more accurately estimated when using more DNAm sites.** DNAm priors are the probability that the DNAm value of selected DNAm sites fall within the DNAm bins 0-0.05, 0.05 - 0.95 and 0.95 – 1, shown in maroon, green and blue, respectively. Priors were calculated across 125 samples. The true value for each prior, calculated across the entire dataset, is shown in red.

**Supplementary Figure 6: The proportion of DNAm sites remaining is more accurately estimated when using more DNAm sites.** The proportion of DNAm sites remaining after filtering by minimum read depth and minimum number of samples calculated from an increasing number of DNAm sites from an RRBS dataset. n was calculated across 125 samples. The red dashed line is the true value of n, calculated from the entire dataset.

**Supplementary Table 1**: **A summary of parameters used in simulation analysis.**

| **Plot** | **μRD** | **N_1,_ N_2_** | **ΔμDNAm** | **μDNAm** |
| --- | --- | --- | --- | --- |
| A | Variable | 30 | 0.2 | 0.25 |
|  |  |  | 0.1 |  |
|  |  |  | 0.05 |  |
| B | 20 | Variable | 0.2 | 0.25 |
|  |  | 5 |  |  |
|  |  | 10 |  |  |
| C | 25 | 20 | Variable | 0.25 |
|  |  | 50 |  |  |
|  |  | 100 |  |  |
|  |  | 500 |  |  |
| D, E | 50 | 80 | 0.05 | Variable |
|  | 30 |  |  |  |
|  | 10 |  |  |  |

Each row refers to a plot in **Figure 6**. Values were chosen so that the variable of interest could be seen to influence power within the scale of the figure.
